# Synthesis of Hierarchical Porous Ni_1.5_Co_1.5_S_4_/g-C_3_N_4_ Composite for Supercapacitor with Excellent Cycle Stability

**DOI:** 10.3390/nano10091631

**Published:** 2020-08-20

**Authors:** Fangzhou Jin, Xingxing He, Jinlong Jiang, Weijun Zhu, Jianfeng Dai, Hua Yang

**Affiliations:** 1 Department of Physics, School of Science, Lanzhou University of Technology, Lanzhou 730050, China; jfz1995@163.com (F.J.); 15641675540@163.com (X.H.); zhuwj2020@163.com (W.Z.); daijf@lut.edu.cn (J.D.); hyang@lut.edu.cn (H.Y.); 2 State Key Laboratory of Advanced Processing and Recycling of Nonferrous Metals, Lanzhou University of Technology, Lanzhou 730050, China

**Keywords:** g-C_3_N_4_, metal sulfide, supercapacitor, cycle stability

## Abstract

In this work, the hierarchical porous Ni_1.5_Co_1.5_S_4_/g-C_3_N_4_ composite was prepared by growing Ni_1.5_Co_1.5_S_4_ nanoparticles on graphitic carbon nitride (g-C_3_N_4_) nanosheets via a hydrothermal route. Due to the self-assembly of larger size g-C_3_N_4_ nanosheets as a skeleton, the prepared nanocomposite possesses a unique hierarchical porous structure that can provide short ions diffusion and fast electron transport. As a result, the Ni_1.5_Co_1.5_S_4_/g-C_3_N_4_ composite exhibits a high specific capacitance of 1827 F g^−1^ at a current density of 1 A g^−1^, which is 1.53 times that of pure Ni_1.5_Co_1.5_S_4_ (1191 F g^−1^). In particular, the Ni_1.5_Co_1.5_S_4_/g-C_3_N_4_//activated carbon (AC) asymmetric supercapacitor delivers a high energy density of 49.0 Wh kg^−1^ at a power density of 799.0 W kg^−1^. Moreover, the assembled device shows outstanding cycle stability with 95.5% capacitance retention after 8000 cycles at a high current density of 10 A g^−1^. The attractive performance indicates that the easily synthesized and low-cost Ni_1.5_Co_1.5_S_4_/g-C_3_N_4_ composite would be a promising electrode material for supercapacitor application.

## 1. Introduction

Supercapacitor has attracted great attention in recent years, due to its high-power density, excellent cycling stability, fast charge-discharge and environmental friendliness [1]. The electrode materials for supercapacitor application mainly include carbon materials [2], metal oxides [3], conductive polymers [4], transition metal sulfides [5,6], and their composites [7]. Among various electrode materials, transition metal sulfides have a broad application prospect because of its inherent characteristics and excellent electrochemical performance [8]. Compared with oxide counterparts, the transition metal sulfides possessed better electrical conductivity, richer electrochemical activity and higher theoretical capacitance. Furthermore, ternary Ni-Co-S sulfides such as NiCo_2_S_4_ and Ni_2_CoS_4_ have been demonstrated to be more attractive than corresponding binary Ni or Co sulfides (e.g., NiS, CoS, Ni_3_S_4_, ect.) [9,10,11,12,13], thanks to their rich redox reaction sites and the advantage in terms of electronic conductivity [14]. Recently, several groups have reported that the atomic ratio of nickel and cobalt plays an important role in optimizing the electrochemical performance of electrodes [15,16,17]. The nonstoichiometric Ni_1.5_Co_1.5_S_4_ showed a higher specific capacitance, attributing to the synergistic effects of nickel species and cobalt species.

Graphitic carbon nitride (g-C_3_N_4_) is a two-dimensional graphite structure composed of sp^2^-hybridzed carbon and nitrogen atoms [18,19]. The presence of high content nitrogen in g-C_3_N_4_ can enhance the electron-donor property of the carbon matrix, resulting in an improvement the electron transport between the active materials [20,21]. Therefore, g-C_3_N_4_ is considered a promising candidate material for electrochemical applications because of its rapid charge separation and relatively slow charge recombination property in the electron transfer process [22]. Some recent research has revealed that the combination of pseudocapacitive materials and g-C_3_N_4_ can effectively enhance the electrochemical performance of electrode materials for supercapacitor applications. For example, Shi et al. synthesized flower-like Ni(OH)_2_/g-C_3_N_4_ via a facile hydrothermal route. This hybrid structure exhibited a specific capacitance of 505.6 F g^−1^ at a current density of 0.5 A g^−1^ [23]. Dong et al. reported g-C_3_N_4_@Ni(OH)_2_ with interconnect honeycomb nanostructure, which exhibited a high specific capacitance of 1768.7 F g^−1^ as well as a better cycling performance with 84% retentions after 4000 cycles [24]. Guan et al. found that the electrochemical performances of NiCo_2_O_4_/g-C_3_N_4_ were extremely dependent on their morphology. The nanoneedle-assembled NiCo_2_O_4_/g-C_3_N_4_ possessed higher specific capacitance, while nanosheets-assembled NiCo_2_O_4_/g-C_3_N_4_ exhibited a better cycling durability [25]. The hybrid structures of metal sulfides and carbon nanomaterials (such as CNTs and graphene) have attracted much attention for high performance supercapacitor [26]. However, to the best of our knowledge, the combination of nonstoichiometric Ni_1.5_Co_1.5_S_4_ and g-C_3_N_4_ has been rarely reported.

Herein, we report the hierarchical porous Ni_1.5_Co_1.5_S_4_/g-C_3_N_4_ composite by a simple solvothermal method. The prepared composite shows a high specific capacitance of 1827 F g^−1^ owing to interconnecting porous structure assembled by Ni_1.5_Co_1.5_S_4_ nanoparticles and 2D g-C_3_N_4_ nanosheets. More impressively, an asymmetric supercapacitor (denoted as Ni_1.5_Co_1.5_S_4_/g-C_3_N_4_//AC) assembled using the optimized Ni_1.5_Co_1.5_S_4_/g-C_3_N_4_ and activated carbon exhibits great practical application value in energy conversion and storage due to its high energy density and power density, and excellent cycling stability.

## 2. Experimental Section

### 2.1. Preparation of Samples

The g-C_3_N_4_ nanosheets were prepared through a simple improved calcination method as reported in the literature [27]. In brief, 1 g of melamine and 3 g of ammonium chloride were mixed and ground thoroughly in an agate mortar. Then the mixtures were put into a quartz boat and heated at 550 °C with a heat rate of 10 °C min^−1^ for 4 h in a tube furnace. After cooling to room temperature, the yellow g-C_3_N_4_ was obtained. Finally, the g-C_3_N_4_ were washed with deionized water and absolute ethanol several times, and ground into powders for further use.

**Figure 1:**
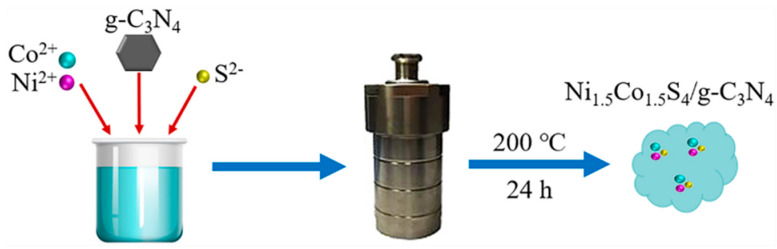
Schematic illustration of the synthesis of Ni_1.5_Co_1.5_S_4_/g-C_3_N_4_.

The Ni_1.5_Co_1.5_S_4_/g-C_3_N_4_ composites were prepared through a modified one-step hydrothermal method as described in our previous paper [28]. Typically, 3 mmol of NiCl_2_6H_2_O, 3 mmol of CoCl_2_6H_2_O and 20 mmol of CS(NH_2_)_2_ were dispersed in a mixture solution of 30 mL water and 50 mL ethylene glycol. Then, 60 mg of g-C_3_N_4_ nanosheets was added to the above solution and stirred magnetically for 30 min. The pH value of the mixed solution was adjusted to 11 using NaOH. Afterwards, the mixed solution was transferred into a 100 mL Teflon-lined stainless-steel autoclave (Xi’an Changyi Instrument Equipment Co., Ltd, Xian, China) and reacted at 200 °C for 24 h. After cooling to ambient temperature, the black precipitates were collected, washed with deionized water and ethanol several times, and dried at 60 °C for 12 h. The preparation process is shown in Figure 1.

### 2.2. Characterizations of Samples

The X-ray diffraction (XRD) patterns of the samples obtained on an X-ray diffractometer (Bruker D8 ADVANCE, Bruker Daltonics Inc., Bruker, Germany) instrument. The X-ray photoelectron spectra (XPS) were collected using a spectrometer (Escalab 250XI, Thermo Fisher Scientific Inc., Walsham, Ma, USA) with monochromatic aluminum target. The morphologies of the samples were observed using a field-emission scanning electron microscope (FESEM, JSM-6701F, JEOL Ltd., Tokyo, Japan) at an accelerating voltage of 5 kV, and a transmission electron microscope (TEM, JEM2010, JEOL Ltd., Tokyo, Japan), respectively. The Brunauer-Emmett-Teller (BET) surface area and Barret-Joyner-Halenda (BJH) pore size distribution of the samples were measured by nitrogen adsorption-desorption isotherms at 77 K using a gas sorption analyzer (Micromeritics ASAP 2020, Micromeritics Instrument Inc., Atlanta, GA, USA).

### 2.3. Electrochemical Measurement

A three-electrode system and two-electrode system were used to test the electrochemical performance of the samples on a CS350H electrochemical workstation with 2 M KOH aqueous as electrolyte. The working electrode was prepared via mixing the active material (2.0 mg, 80 wt.%), Super P conductive carbon black (10 wt.%) and polyvinylidene fluoride binder (10 wt.%). Then, the slurry was coated on a piece of nickel foam current collector (1 cm × 1 cm), and dried at 60 °C for 12 h under vacuum. Finally, the working electrode was fabricated by pressing nickel foam loaded with active material at a pressure of 10 MPa. Platinum plate and saturated Ag/AgCl were used as counter electrode and reference electrode, respectively. An asymmetric supercapacitor (ASC) cell was assembled by using Ni_1.5_Co_1.5_S_4_/g-C_3_N_4_ as the positive electrode and commercial AC as the negative electrode. The electrochemical performance of the electrodes was characterized by cyclic voltammetry (CV) and galvanostatic charge-discharge (GCD) methods. The specific capacitances (C) of the electrodes are calculated based on the GCD curves according to the following Equation [29].

C = (I × ∆t)/(m × ∆V)(1)

where I is the constant discharging current (mA), ∆t is the discharge time (s), the potential window (∆V), and m is the mass of active materials in the electrode (mg). For two-electrode testing, the mass of active materials includes the electroactive materials of both the Ni_1.5_Co_1.5_S_4_/g-C_3_N_4_ and AC.

## 3. Result and Discussion

**Figure 2:**
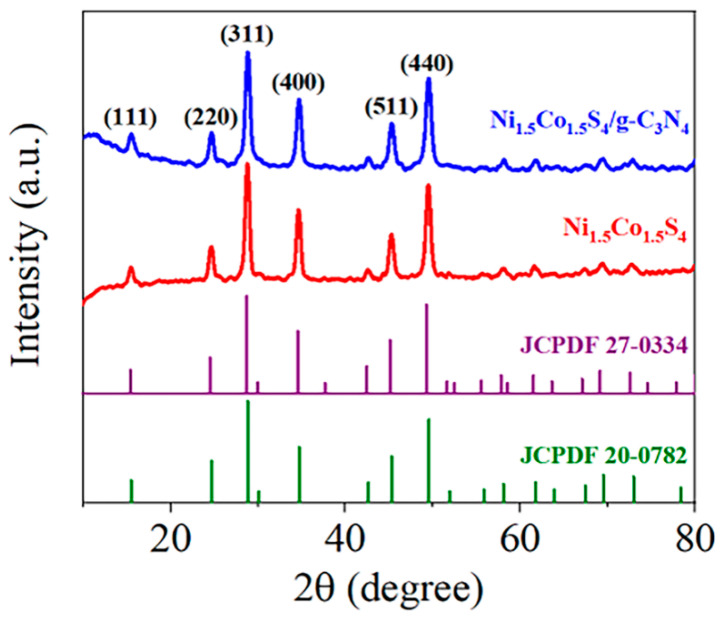
XRD patterns of the Ni_1.5_Co_1.5_S_4_ and Ni_1.5_Co_1.5_S_4_/g-C_3_N_4_.

The phase purity and crystal structure of the samples were analyzed using XRD patterns, and the results are shown in Figure 2. Six diffraction peaks can be perfectly indexed to the (111), (220), (311), (400), (511) and (440) of spinel structured NiCo_2_S_4_ (JCPDS# 20-0782) or Ni_2_CoS_4_ (JCPDS# 24-0334), respectively. In addition, no other metal sulfides such as NiS and Ni_3_S_2_ were observed in the pattern, which indicates the pure spinel structure. Figure S1 shows the XRD pattern of the prepared g-C_3_N_4_. Two diffraction peaks at around 13.1° and 27.3° in g-C_3_N_4_ correspond to the in-plane structure packing of aromatic systems of (100) plane and the interlayer stacking of conjugated aromatic systems of (002) plane, respectively [30,31], which reveals that the prepared g-C_3_N_4_ nanosheets is the typical graphitic structure. No diffraction peaks of g-C_3_N_4_ were found in the XRD pattern of the Ni_1.5_Co_1.5_S_4_/g-C_3_N_4_, which is probably due to weak scattering intensity and relatively low content of g-C_3_N_4_. 

**Figure 3:**
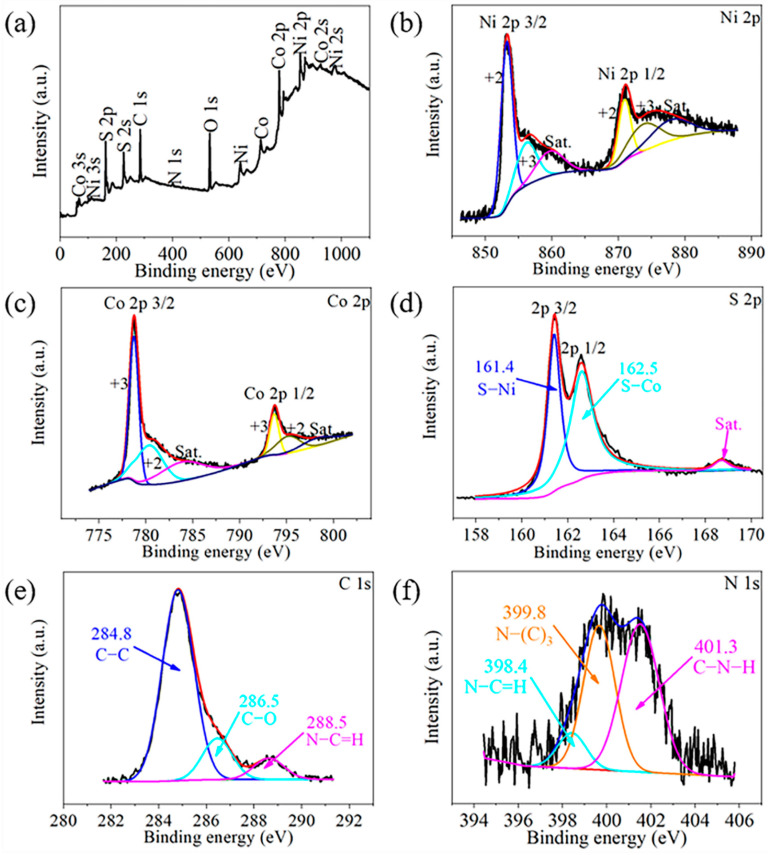
X-ray photoelectron spectra (XPS) of the Ni_1.5_Co_1.5_S_4_/g-C_3_N_4_: (**a**) survey spectrum; (**b**) Ni 2p; (**c**) Co 2p; (**d**) S 2p; (**e**) C 1s and (**f**) N 1s.

In order to determine the chemical bonds of the corresponding elements in the Ni_1.5_Co_1.5_S_4_/g-C_3_N_4_ composite, the XPS spectra of the sample are shown in Figure 3. The XPS survey spectrum (Figure 3a) shows the presence of Ni, Co, S, C, N, and O elements in the sample. The O 1s peak is mainly attributed to contamination when the sample is exposed to ambient air. The high-resolution XPS spectra of Ni 2p, Co 2p, S 2p, C 1s, N 1s are fitted with Gaussian functions to acquire detail information of chemical bonding. For Ni 2p spectrum, the fitting peaks at 853.3 and 856.2 eV are assigned to Ni^2+^ and Ni^3+^, respectively. For Co 2p spectrum, the fitting peaks at 778.7 and 780.9 eV are assigned to Co^3+^ and Co^2+^, respectively. Moreover, two satellite peaks can be observed in each high-resolution Ni 2p and Co 2p spectra. Obviously, the low-valent and high-valent metal ions coexist in the Ni_1.5_Co_1.5_S_4_/g-C_3_N_4_ composite, which is similar to previous reports [28]. Chen et al. believed that the easily valence-changed nickel can contribute the most faradaic capacity of the active materials, while the low-valent cobalt can offer the high electronic conductivity and assist the charge-transfer process in the binary metal sulfides based active materials [17]. Two peaks S 2p (Figure 3d) located at binding energy of 161.4 and 162.5 eV are typical of metal-sulfur bonds [32,33]. The C 1s spectrum (Figure 3e) is fitted into three peaks which could be attributed to sp^2^ C–C (284.8 eV), C–O (286.5 eV) and N–C=N or C–(N)_3_ (288.5 eV) bonds, respectively [22]. Figure 3f shows the three different kinds of chemical states of nitrogen species in the g-C_3_N_4_. According to the literature [34,35,36], the peaks at binding energy of 398.4, 399.8 and 401.3 eV are assigned to sp^2^ nitrogen in carbon containing triazine rings (C=N–C), bridged graphitic tertiary nitrogen bonded with carbon atom (N–(C)_3_), and amino functional groups (C–N–H), respectively. These peaks are agreement with the characteristics of nitrogen species in g-C_3_N_4_. 

**Figure 4:**
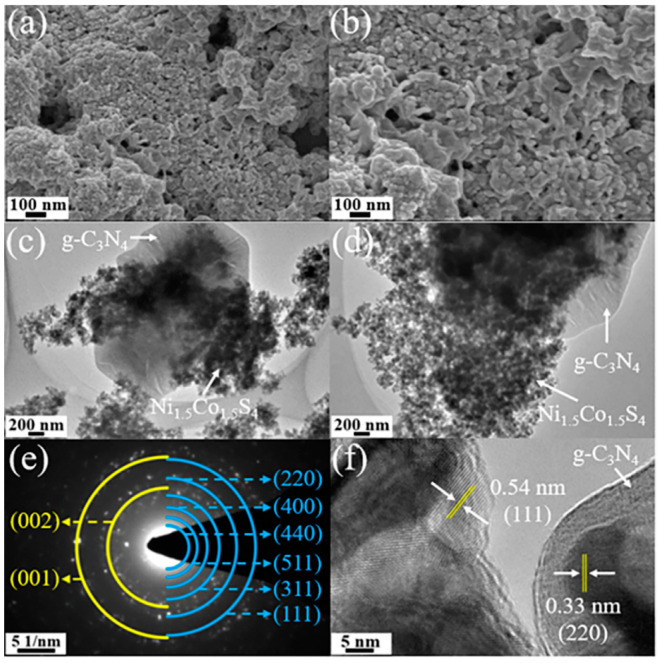
(**a**,**b**) SEM images; (**c**,**d**) TEM images; (**e**) SAED (selected area electron diffraction) pattern and (**f**) TEM image of the Ni_1.5_Co_1.5_S_4_/g-C_3_N_4_.

**Figure 5:**
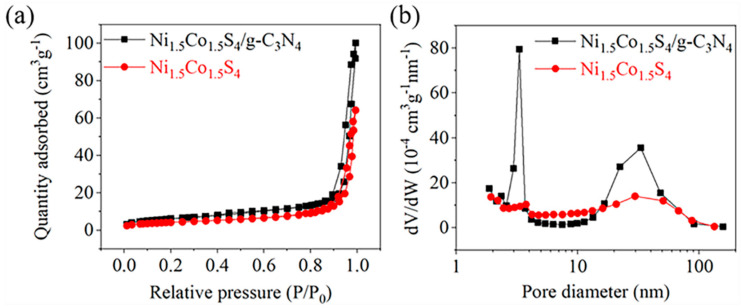
(**a**) Nitrogen adsorption-desorption isotherm and (**b**) Pore-size distribution curves of the Ni_1.5_Co_1.5_S_4_ and the Ni_1.5_Co_1.5_S_4_/g-C_3_N_4_.

Figure 4a,b shows the morphology of the Ni_1.5_Co_1.5_S_4_/g-C_3_N_4_. The as-prepared composite is composed of g-C_3_N_4_ nanosheets and Ni_1.5_Co_1.5_S_4_ nanoparticles. Compared with the Ni_1.5_Co_1.5_S_4_ (Figure S2), some macroporous structure is clearly observed in the Ni_1.5_Co_1.5_S_4_/g-C_3_N_4_ due to self-assemble of larger size g-C_3_N_4_ nanosheets as skeleton. The Ni_1.5_Co_1.5_S_4_/g-C_3_N_4_ has higher porosity which is also confirmed by the gas sorption experiments in Figure 5. It is seen from TEM images (Figure 4c,d) that a large number of Ni_1.5_Co_1.5_S_4_ nanoparticles (30–60 nm) were anchored on the on the surface of g-C_3_N_4_ nanosheets (0.8–2.0 μm). The selected area electron diffraction (SAED) pattern displays two sets of diffraction rings that can be indexed to the graphic structure g-C_3_N_4_ (yellow rings) and the spinel structure Ni_1.5_Co_1.5_S_4_ (blue rings), respectively. The high-resolution transmission electron microscope (HRTEM) image shows the formation of the distinct nanoparticle-on-nanosheet heterostructure.

The pore structures of the Ni_1.5_Co_1.5_S_4_ and the Ni_1.5_Co_1.5_S_4_/g-C_3_N_4_ were tested by nitrogen adsorption-desorption at 77 K. As shown in Figure 5a, the samples display type IV isotherm with typical H1 hysteresis loop at a relative pressure of 0.8–1.0, which is characteristic for mesoporous materials [37]. The BET specific surface area of the Ni_1.5_Co_1.5_S_4_/g-C_3_N_4_ is 22.5 m^2^ g^−1^, which is much higher than that of the Ni_1.5_Co_1.5_S_4_ (15.2 m^2^ g^−1^). It is seen from Figure 5b that two samples are mainly composed of mesoporous and macrospores, suggesting a hierarchical porous structure (Table S1). The BJH desorption cumulative volume of pores between 1.7 nm and 300.0 nm notably increases from 0.100 cm^3^ g^−1^ for Ni_1.5_Co_1.5_S_4_ to 0.124 cm^3^ g^−1^ for the Ni_1.5_Co_1.5_S_4_/g-C_3_N_4_, while the average pore diameter slightly decreases from 25.5 nm to 24.9 nm. These results indicate that the addition of g-C_3_N_4_ can not only increase the specific surface area, but also optimize the structure of pores. Consequently, an increase of the mesoporous channels in the Ni_1.5_Co_1.5_S_4_/g-C_3_N_4_ is more beneficial for the fast ion transportation to improve the electrochemical activity of the electrodes. 

**Figure 6:**
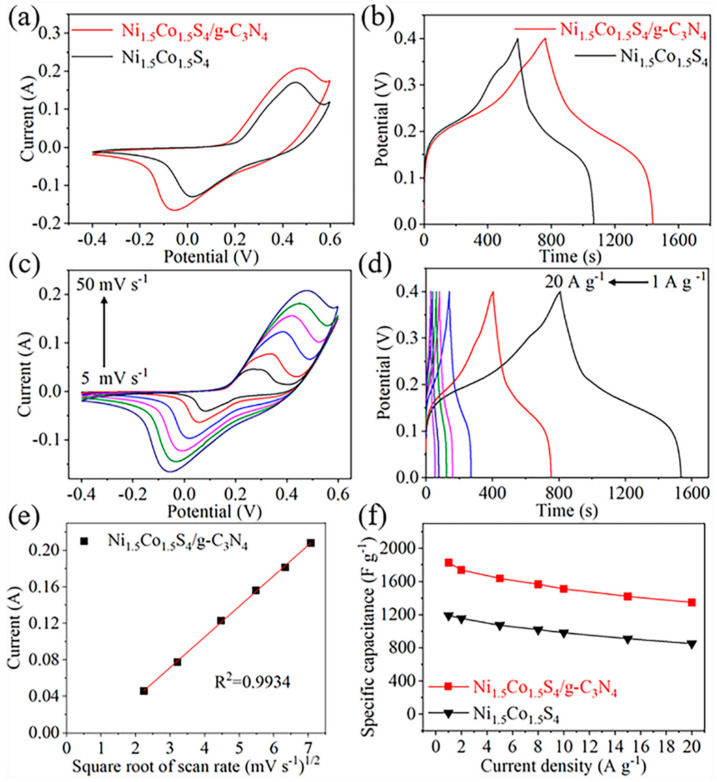
Electrochemical properties of the Ni_1.5_Co_1.5_S_4_ and the Ni_1.5_Co_1.5_S_4_/g-C_3_N_4_: (**a**,**c**) cyclic voltammetry (CV) curves; (**b**,**d**) galvanostatic charge-discharge (GCD) curves; (**e**) the linear relation between the anodic peak current and square root of scan rate; (**f**) the specific capacitance at different current densities.

Figure 6 shows electrochemical properties of the samples tested through the three-electrode system. The CV curves was performed at a scan rate of 50 mV s^−1^ within potential window of −0.4–0.6 V. As shown in Figure 6a, the redox peaks of the Ni_1.5_Co_1.5_S_4_/g-C_3_N_4_ is similar to those of the Ni_1.5_Co_1.5_S_4_, which can be attributed to the reversible process of Ni^2+^/Ni^3+^ and Co^2+^/Co^3+^ associated with the insertion and extraction of OH^−^ anions to and from the electrode materials [38]. The integral area of the CV loop of the Ni_1.5_Co_1.5_S_4_/g-C_3_N_4_ is larger than that of the Ni_1.5_Co_1.5_S_4_, indicating superior electrochemical performance. This result can be further confirmed by GCD tests in Figure 6b. Figure 6c,d show the CV and GCD curves of the Ni_1.5_Co_1.5_S_4_/g-C_3_N_4_ at different scan rates and current densities. They almost maintain the symmetric shape without visible distort, suggesting that the electrode has excellent pseudocapacitive behavior and high coulombic efficiency. The anodic peak current shows a linear relationship with the square root of scan rate (Figure 6e), which indicates that the electrochemical kinetics is a diffusion-controlled process. The specific capacitances of the samples were calculated at the current densities ranging from 1 A g^−1^ to 20 A g^−1^ according to the GCD curves. The Ni_1.5_Co_1.5_S_4_/g-C_3_N_4_ composite exhibits a high specific capacitance of 1827 F g^−1^ at a current density of 1 A g^−1^ (Figure 6f), which is 1.53 times that of the Ni_1.5_Co_1.5_S_4_ (1191 F g^−1^). Even if the current density increases 20 times, the specific capacitance still reaches to 1348 F g^−1^, demonstrating a good rate performance. This result is superior to those of the most recently reported composites such as Ni–Co–S/graphene and NiCo_2_S_4_@g-C_3_N_4_ composites [36,39]. Moreover, the CV and GCD curves of pure g-C_3_N_4_ nanosheets is shown Figure S3 for a comparation. The specific capacitance of g-C_3_N_4_ nanosheets is only 11 F g^−1^ at a current density of 1 A g^−1^, which is far lower than that of the Ni_1.5_Co_1.5_S_4_/g-C_3_N_4_ composite. In order to further explore the effect of g-C_3_N_4_ content on the electrochemical properties, the Ni_1.5_Co_1.5_S_4_/g-C_3_N_4_ composites with different g-C_3_N_4_ content were also prepared and evaluated, shown in Figure S4. When the amount of g-C_3_N_4_ is 60 mg, the Ni_1.5_Co_1.5_S_4_/g-C_3_N_4_ composite shows the highest specific capacitance, owing to maximizing synergetic effects of Ni_1.5_Co_1.5_S_4_ nanoparticles and g-C_3_N_4_ nanosheets. However, the specific capacitance decreases when 90 mg of g-C_3_N_4_ is introduced. This superior supercapacitive performance of the Ni_1.5_Co_1.5_S_4_/g-C_3_N_4_ can be mainly ascribed to two reasons. On the one hand, g-C_3_N_4_ nanosheets can increase the specific surface area and mesoporous number, which provides more active sites for interface reaction and shortens the pathway of the electrolyte ion diffusion. On the other hand, g-C_3_N_4_ nanosheets can improve electrical conductivity of the Ni_1.5_Co_1.5_S_4_, which facilitates for electron transport. As shown in Figure S5, the impedance plots imply that the Ni_1.5_Co_1.5_S_4_/g-C_3_N_4_ composite possesses smaller internal resistance, faster ion diffusion process and lower charge transfer resistance during the faradic reaction.

**Figure 7:**
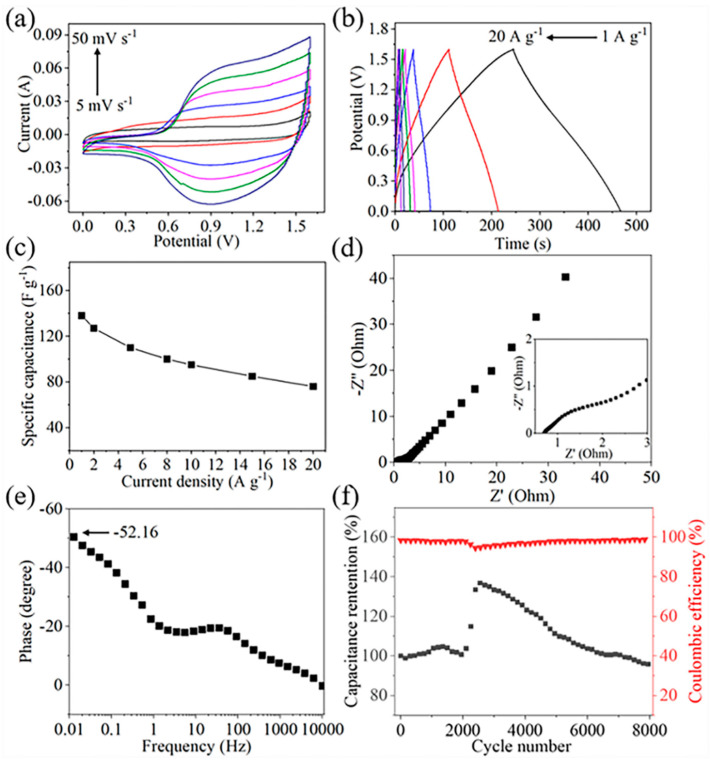
Electrochemical characterizations of the Ni_1.5_Co_1.5_S_4_/g-C_3_N_4_//AC supercapacitor (**a**) CV curves; (**b**) GCD curves; (**c**) specific capacitance at different current densities; (**d**) Nyquist plot; (**e**) plot of phase angle verses frequency; (**f**) cycling stability at a current density of 10 Ag^−1^.

**Figure 8:**
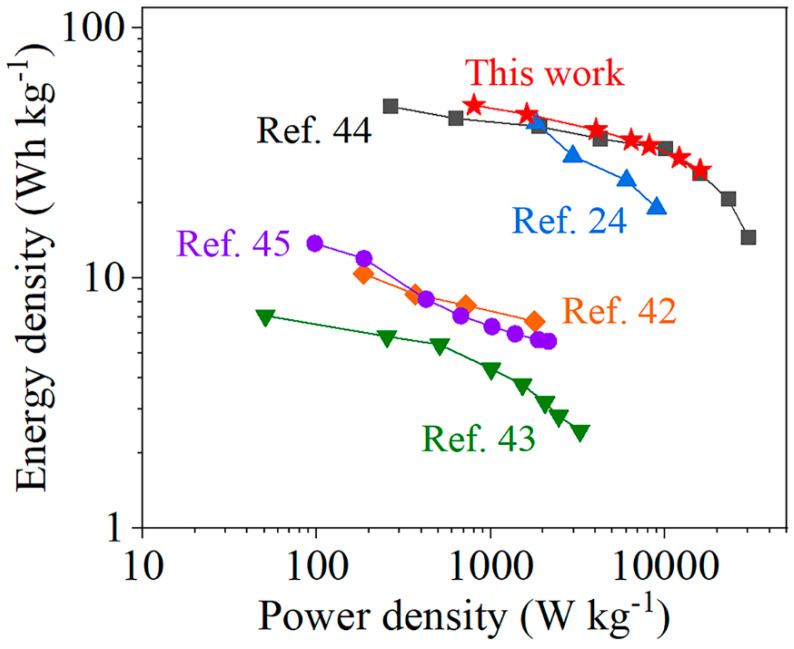
Ragone plot of energy density and power density.

Figure 7 shows the performance of the Ni_1.5_Co_1.5_S_4_/g-C_3_N_4_//AC supercapacitor. The working voltage window of the device was extended to 1.6 V (Figure 7a), because the potential window of the Ni_1.5_Co_1.5_S_4_/g-C_3_N_4_ and AC is in the range of −0.4 to 0.6 V and −1 to 0 V, respectively. Apparently, the capacitance of the device comes from the combined contribution of pseudocapacitive and electrical double behaviors. Furthermore, the charge-discharge curves are good symmetric with a coulombic efficiency of over 98.0% at different scan rate, demonstrating its high electrochemical reversibility (Figure 7b). The specific capacitance of the device is calculated to be 138 F g^−1^ at 1 A g^−1^, and it still retains 76 F g^−1^ even at a high current density of at 20 A g^−1^ (Figure 7c). Figure 7d shows the Nyquist plot of device in the frequency range of 10^−2^ to 10^5^ Hz. The equivalent series resistance (Rs) and the charge transfer resistance (Rct) are as low as 0.73 and 1.55 Ω, respectively, which are considered to be good for improved charge-discharge rate and power density of the device. The impedance phase angle of the device is approximately −52.16° at a frequency of 0.01 Hz, and reaches −45° at a frequency of 0.04 Hz (Figure 7e). The resistance and reactance of the capacitor have equal magnitudes at the phase angle of −45°, so the frequency at this point is convenient for comparison [40]. This frequency of the Ni_1.5_Co_1.5_S_4_/g-C_3_N_4_//AC device is comparable to that of an activated carbon-based electric double-layer capacitor (0.05 Hz) [41]. Figure 7f shows the cycling stability of the device at a current density of 10 A g^−1^. After 8000 cycles, the capacitance retention and the columbic efficiency still kept about 95.5% and 98.4%, respectively, indicating outstanding long-term stability. Energy density (E) and power density (P) are used as two major parameters to evaluate the performance of supercapacitor in practical applications [31]. Figure 8 shows a Ragone plot of energy density and power density. The Ni_1.5_Co_1.5_S_4_/g-C_3_N_4_//AC supercapacitor delivers high energy density of 49.0 Wh kg^−1^ at a power density of 799.0 W kg^−1^. These values surpass those of previously reported symmetric and asymmetric supercapacitors based on g-C_3_N_4_ composites, such as g-C_3_N_4_@Ni(OH)_2_ [24], ZnS/g-C_3_N_4_ [42] and porous g-C_3_N_4_ [43,44,45].

## 4. Conclusions

In summary, we have prepared the hierarchical porous Ni_1.5_Co_1.5_S_4_/g-C_3_N_4_ composite by growing Ni_1.5_Co_1.5_S_4_ nanoparticles on g-C_3_N_4_ nanosheets using a hydrothermal method. Compared with pure Ni_1.5_Co_1.5_S_4_, the Ni_1.5_Co_1.5_S_4_/g-C_3_N_4_ composite possesses larger surface area and optimized porous structures. The specific capacitance of the composites is strongly depended on the content of g-C_3_N_4_ nanosheets. When the adding amount of g-C_3_N_4_ is 60 mg, the Ni_1.5_Co_1.5_S_4_/g-C_3_N_4_ composite exhibits the highest specific capacitance of 1827 F g^−1^ at a current density of 1 A g^−1^, which is 1.53 times that of pure Ni_1.5_Co_1.5_S_4_. The enhancement in specific capacitance could be attributed to maximizing synergetic effects of Ni_1.5_Co_1.5_S_4_ nanoparticles and g-C_3_N_4_ nanosheets. A Ni_1.5_Co_1.5_S_4_/g-C_3_N_4_//AC asymmetric supercapacitor exhibits a high energy density of 49.0 Wh kg^−1^ at a power density of 799.0 W kg^−1^, and outstanding cycle stability with 95.5% capacitance retention after 8000 cycles at a current density of 10 A g^−1^.

## Supplementary Materials

The following are available online at https://www.mdpi.com/2079-4991/10/9/1631/s1, Figure S1: XRD pattern of g-C_3_N_4_, Figure S2: SEM images of pure Ni_1.5_Co_1.5_S_4_, Figure S3: Electrochemical properties of the Ni_1.5_Co_1.5_S_4_@g-C_3_N_4_ with different content of g-C_3_N_4_: (a) CV curves, (b) GCD curves, Figure S4: Electrochemical properties of the Ni_1.5_Co_1.5_S_4_/g-C_3_N_4_ with different content of g-C_3_N_4_: (a) CV curves, (b) GCD curves, (c) the specific capacitance at different current densities, and (d) the dependence of specific capacitance on g-C_3_N_4_ content, Figure S5: Nyquist plot of pure Ni_1.5_Co_1.5_S_4_ and Ni_1.5_Co_1.5_S_4_@g-C_3_N_4_, Table S1: The cumulative pore volume of Ni_1.5_Co_1.5_S_4_ and Ni_1.5_Co_1.5_S_4_@g-C_3_N_4_.

## References

[1] Nguyen V.H., Shim J.J. (2015). In Situ Growth of Hierarchical Mesoporous NiCo_2_S_4_@MnO_2_ Arrays on Nickel Foam for High-Performance Supercapacitors. Electrochim. Acta.

[2] Azadmanjiri J., Srivastava V.K., Kumar P., Nikzad M., Wang J., Yu A. (2018). Two- and Three-Dimensional Graphene-Based Hybrid Composites for Advanced Energy Storage and Conversion Devices. J. Mater. Chem. A..

[3] Davoglio R.A., Cabello G., Marco J.F., Biaggio S.R. (2018). Synthesis and Characterization of α-MnO_2_ Nanoneedles for Electrochemical Supercapacitors. Electrochim. Acta.

[4] Tan S.C., Li J.J., Zhou L.J., Chen P., Shi J.T., Xu Z.Y. (2018). Modified Carbon Fiber Paper-Based Electrodes Wrapped by Conducting Polymers with Enhanced Electrochemical Performance for Supercapacitors. Polymers.

[5] Peng S.J., Li L.L., Li C.C., Tan H.T., Cai R., Yu H., Mhaisalkar S., Srinivasan M., Ramakrishna S., Yan Q. (2013). In Situ Growth of NiCo_2_S_4_ Nanosheets on Graphene for High-Performance Supercapacitors. Chem. Commun..

[6] Liang K.Q., He W.D., Deng X.L., Ma H., Xu X.J. (2018). Controlled Synthesis of NiCo_2_S_4_ Hollow Spheres as High-Performance Electrode Materials for Supercapacitors. J. Alloys Compd..

[7] Bi T.T., Jiang J.L., Lei Y., Zheng X., Jia Z.F., Wei Z.Q., Yang H. (2020). Improving Supercapacitive Performance of CNTs/NiCo_2_S_4_ Composites by Interface Regulation. Appl. Surf. Sci..

[8] Guan B.Y., Yu L., Wang X., Song S.Y., Lou X.W. (2017). Formation of Onion-Like NiCo_2_S_4_ Particles Via Sequential Ion-Exchange for Hybrid Supercapacitors. Adv. Mater..

[9] Beka L.G., Li X., Xia X.J., Liu W.H. (2017). 3D Flower-Like CoNi_2_S_4_ Grown on Graphene Decorated Nickel Foam as High Performance Supercapacitor. Diam. Relat. Mater..

[10] Guan B., Li Y., Yin B.Y., Liu K.F., Wang D.W., Zhang H.H. (2017). Synthesis of Hierarchical NiS Microflowers for High Performance Asymmetric Supercapacitor. Chem. Eng. J..

[11] Zhang Y.H., Lv C.X., Wang X., Chen S., Li D.H., Peng Z., Yang D.J. (2018). Boosting Sodium-Ion Storage by Encapsulating NiS(CoS) Hollow Nanoparticles into Carbonaceous Fibers. ACS Appl. Mater. Interfaces.

[12] Dai S., Zhao B., Qu C., Chen D.C., Dang D., Song B., DeGlee B.M., Fu J.W., Hu C.G., Wong C.-P. (2017). Controlled Synthesis of Three-Phase Ni_x_S_y_/rGO Nanoflake Electrodes for Hybrid Supercapacitors with High-Energy and Power Density. Nano Energy.

[13] Chen H.C., Jiang J.J., Zhang L., Wan H.Z., Qi T., Xia D.D. (2013). Highly Conductive NiCo_2_S_4_ Urchin-Like Nanostructures for High-Rate Pseudocapacitors. Nanoscale.

[14] Xia C., Li P., Gandi A.N., Schwingenshl€ogl U., Alshareef H.N. (2015). Is NiCo_2_S_4_ Really a Semiconductor?. Chem. Mater..

[15] Chen H.C., Jiang J.J., Zhao Y.D., Zhang L. (2015). One-Pot Synthesis of Porous Nickel Cobalt Sulphides: Tuning the Composition for Superior Pseudocapacitance. J. Mater. Chem. A.

[16] Zhang L., Zhang H.T., Jin L., Zhang B.B., Liu F.Y., Su H., Chun F.J., Li Q.H., Peng J.F., Yang W.Q. (2016). Composition Controlled Nickel Cobalt Sulfide Score-Shell Structure as High Capacity and Good Rate-Capability Electrodes for Hybrid Supercapacitors. RSC Adv..

[17] Chen T., Tang Y.F., Guo W.F., Qiao Y.Q., Yu S.X., Mu S.C., Wang L., Zhao Y.F., Gao F. (2016). Synergistic Effect of Cobalt and Nickel on the Superior Electrochemical Performances of rGO Anchored Nickel Cobalt Binary Sulfides. Electrochim. Acta.

[18] Babu B., Koutavarapu R., Shim J., Yoo K. (2020). Enhanced visible-light-driven photoelectrochemical and photocatalytic performance of Au-SnO_2_ quantum dot-anchored g-C_3_N_4_ nanosheets. Sep. Pur. Technol..

[19] Koutavarapu R., Babu B., Reddy C.V., Yoo K., Cho M., Shim J. (2010). A novel one-pot approach of ZnWO_4_ nanorods decorated onto g-C_3_N_4_ nanosheets: 1D/2D heterojunction for enhanced solar-light-driven photocatalytic activity. J. Mater. Sci..

[20] Li X.D., Feng Y., Li M.C., Li W., Wei H., Song D.D. (2015). Smart Hybrids of Zn_2_GeO_4_ Nanoparticles and Ultrathin g-C_3_N_4_ Layers: Synergistic Lithium Storage and Excellent Electrochemical Performance. Adv. Funct. Mater..

[21] Chen A.Y., Zhang T.T., Qiu Y.J., Wang D., Wang P., Li H.J., Li Y., Yang J.H., Wang X.Y., Xie X.F. (2019). Construction of Nanoporous Gold/g-C_3_N_4_ Heterostructure for Electrochemical Supercapacitor. Electrochim. Acta.

[22] Thiagarajan K., Bavani T., Arunachalam P., Lee S.J., Theerthagiri J., Madhavan J., Pollet B.G., Choi M.Y. (2020). Nanofiber NiMoO_4_/g-C_3_N_4_ Composite Electrode Materials for Redox Supercapacitor Applications. Nanomaterials.

[23] Shi L., Zhang J.L., Liu H.D., Que M.N., Cai X., Tan S.Z., Huang L.H. (2015). Flower-Like Ni(OH)_2_ Hybridized g-C_3_N_4_ for High-Performance Supercapacitor Electrode Material. Mater. Lett..

[24] Dong B., Li M.Y., Chen S., Ding D.W., Wei W., Gao G.X., Ding S.J. (2017). Formation of g-C_3_N_4_@Ni(OH)_2_ Honeycomb Nanostructure and Asymmetric Supercapacitor with High Energy and Power Density. ACS Appl. Mater. Interfaces.

[25] Guan B., Shan Q.Y., Chen H., Xue D.F., Chen K.F., Zhang Y.X. (2016). Morphology Dependent Supercapacitance of Nanostructured NiCo_2_O_4_ on Graphitic Carbon Nitride. Electrochim. Acta.

[26] Halim M., Liu G., Ardhi R.E.A., Hudaya C., Wijaya O., Lee S.-H., Kim A.-Y., Lee J.K. (2017). Pseudocapacitive characteristics of low-carbon silicon oxycarbide for lithium-ion capacitors. ACS Appl. Mater. Interfaces.

[27] Li Q., Xu D., Guo J.N., Ou X., Yan F. (2017). Protonated g-C_3_N_4_@Polypyrrole Derived N-doped Porous Carbon for Supercapacitors and Oxygen Electrocatalysis. Carbon.

[28] He X.X., Bi T.T., Zheng X., Zhu W.J., Jiang J.L. (2020). Nickel Cobalt Sulfide Nanoparticles Grown on Titanium Carbide MXenes for High-Performance Supercapacitor. Electrochim. Acta.

[29] Zhang L.G., Chen X.F., Guan J., Jiang Y.J., Hou T.G., Mu X.D. (2013). Facile Synthesis of Phosphorus Doped Graphitic Carbon Nitride Polymers with Enhanced Visible-Light Photocatalytic Activity. Mater. Res. Bull..

[30] Wan W., Sun J.Y., Ye S., Zhang Q.Y. (2018). Confining the Polymerization Degree of Graphitic Carbon Nitride in Porous Zeolite-Y and Its Luminescence. RSC Adv..

[31] Ding Y.B., Tang Y.H., Yang L.M., Zeng Y.X., Yuan J.L., Liu T., Zhang S.Q., Liu C.B., Luo S.L. (2016). Porous Nitrogen-Rich Carbon Materials from Carbon Self Repairing g-C_3_N_4_ Assembled with Graphene for High Performance Supercapacitor. J. Mater. Chem. A.

[32] Adhikari S.P., Awasthi G.P., Kim K.-S., Park C.H., Kim C.S. (2018). Synthesis of Three-Dimensional Mesoporous Cu-Al Layered Double Hydroxide/g-C_3_N_4_ Nanocomposites on Ni-Foam for Enhanced Supercapacitors with Excellent Long-Term Cycling Stability. Dalton Trans..

[33] Wang D.Z., Zhu W.L., Yuan Y., Du G., Zhu J.L., Zhu X.H., Pezzotti G. (2018). Kelp-Like Structured NiCo_2_S_4_-C-MoS_2_ Composite Electrodes for High Performance Supercapacitor. J. Alloys Compd..

[34] Yu J.G., Wang S.H., Cheng B., Lin Z., Huang F. (2013). Noble Metal-Free Ni(OH)_2_–g-C_3_N_4_ Composite Photocatalyst with Enhanced Visible-Light Photocatalytic H_2_-Production Activity. Catal. Sci. Technol..

[35] He G., Qiao M., Li W.Y., Lu Y., Zhao T.T., Zou R.J., Li B., Darr J.A., Hu J.Q., Titirici M.-M. (2017). S,N-Co-doped Graphene-Nickel Cobalt Sulfide Aerogel: Improved Energy Storage and Electrocatalytic Performance. Adv. Sci..

[36] Guo W., Wang J.Y., Fan C., Chen Z., Liu P., Zhu D.J., Xu Z.L., Pang L., Li T. (2017). Synthesis of Carbon Self-Repairing Porous g-C_3_N_4_ Nanosheets/NiCo_2_S_4_ Nanoparticles Hybrid Composite as High-Performance Electrode Materials for Supercapacitors. Electrochim. Acta.

[37] Rouquerol J., Avnir D., Fairbridge C.W., Everett D.H., Haynes J.H., Pernicone N., Ramsay J.D.F., Sing K.S.W., Unger K.K. (1994). Recommendations for the Characterization of Porous Solids Pure. Appl. Chem..

[38] Ensafi A.A., Abarghoui M.M., Rezaei B. (2019). Graphitic Carbon Nitride Nanosheets Coated with Ni_2_CoS_4_ Nanoparticles as a High-Rate Electrode Material for Supercapacitor Application. Ceram. Int..

[39] Yang J., Yu C., Fan X.M., Liang S.X., Li S.F., Huang H.W., Ling Z., Hao C., Qiu J.S. (2016). Electroactive Edge Site-Enriched Nickel-Cobalt Sulfide into Graphene Frameworks for High-Performance Asymmetric Supercapacitors. Energy Environ. Sci..

[40] Sheng K.X., Sun Y.Q., Li C., Yuan W.J., Shi G.Q. (2012). Ultrahigh-Rate Supercapacitors Based on Eletrochemically Reduced Graphene Oxide for Ac Line-Filtering. Sci. Rep..

[41] Feng J., Sun X., Wu C.Z., Peng L.L., Lin C.W., Hu S.L., Yang J.L., Xie Y. (2011). Metallic Few-Layered VS_2_ Ultrathin Nanosheets: High Two-Dimensional Conductivity for In-Plane Supercapacitors. J. Am. Chem. Soc..

[42] Wei B.B., Liang H.F., Wang R.R., Zhang D.F., Qi Z.B., Wang Z.C. (2018). One-Step Synthesis of Graphitic-C_3_N_4_/ZnS Composites for Enhanced Supercapacitor Performance. J. Energy Chem..

[43] Wang D.H., Wang Y.Z., Chen Y., Liu W., Wang H.Q., Zhao P.H., Li Y., Zhang J.F., Dong Y.G., Hu S.L. (2018). Coal Tar Pitch Derived N-doped Porous Carbon Nanosheets by the In-Situ Formed g-C_3_N_4_ as a Template for Supercapacitor Electrodes. Electrochim. Acta.

[44] Chen T., Wei S.T., Wang Z.H. (2020). NiCo_2_S_4_ Based Composite Materials for Supercapacitors. ChemPlusChem.

[45] Liu L., Liu A.R., Xu Y.H., Yang F.Q., Wang J., Deng Q., Zeng Z.L., Deng S.G. (2020). Fabrication of dual-hollow heterostructure of Ni_2_CoS_4_ sphere and nanotubes as advanced electrode for high-performance flexible all-solid-state supercapacitors. J. Colloid Interf. Sci..

