# Supplementary material for: Synthesis of Hierarchical Porous Ni1.5Co1.5S4/g-C3N4 Composite for Supercapacitor with Excellent Cycle Stability
Source: Nanomaterials (Basel). 2020 Aug 20;10(9):1631. doi: 10.3390/nano10091631 (PMC7558685; doi:10.3390/nano10091631)
Supplement: Supplementary file 1 [file nanomaterials-10-01631-s001.pdf]

Supplementary Material

Synthesis of hierarchical porous $Ni_{1.5}Co_{1.5}S_4$/g-C_3N_4 composite for supercapacitor with excellent cycle stability

Fangzhou Jin[1], Xingxing He[1], Jinlong Jiang[1,2*], Weijun Zhu[1], Jianfeng Dai, Hua Yang[1]

[1] Department of Physics, School of Science, Lanzhou University of Technology, Lanzhou 730050, PR China

[2] State Key Laboratory of Advanced Processing and Recycling of Nonferrous Metals, Lanzhou University of Technology, Lanzhou 730050, PR China

Correspondence: golden_dragon@126.com (J. Jiang)

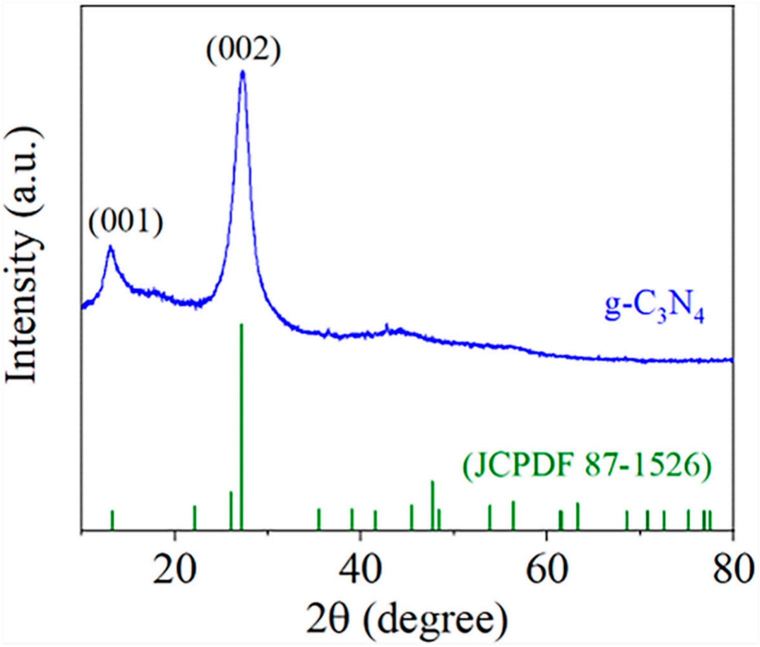

Figure S1 XRD pattern of g-C_3N_4

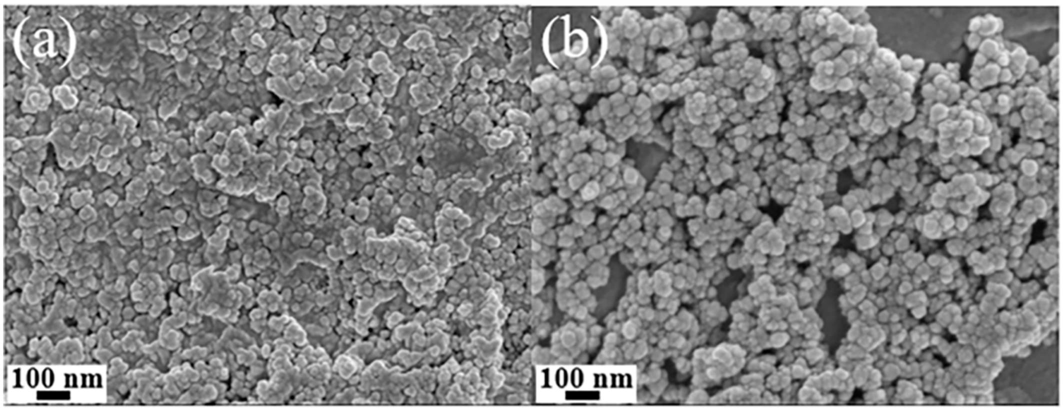

Figure S2 SEM images of pure $Ni_{1.5}Co_{1.5}S_4$

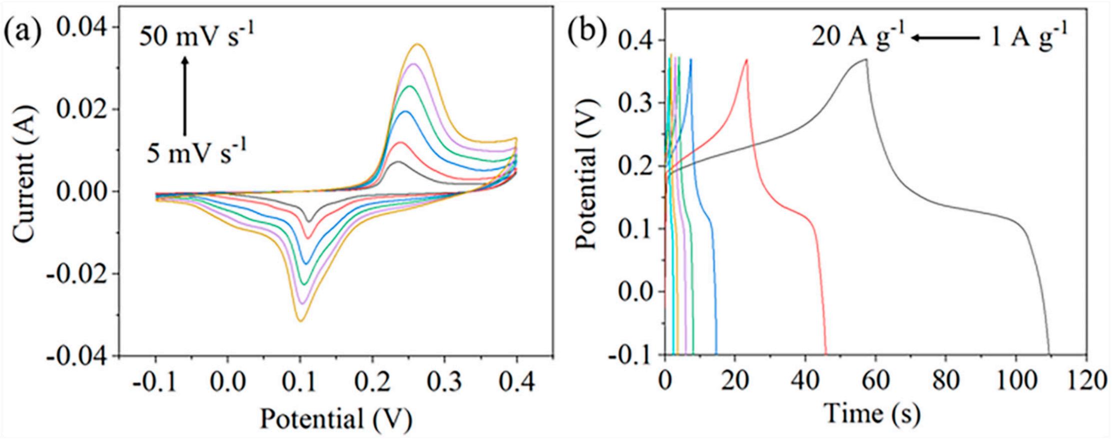

Figure S3 Electrochemical characterizations of the g-C_3N_4 (a) CV curves; (b) GCD curves

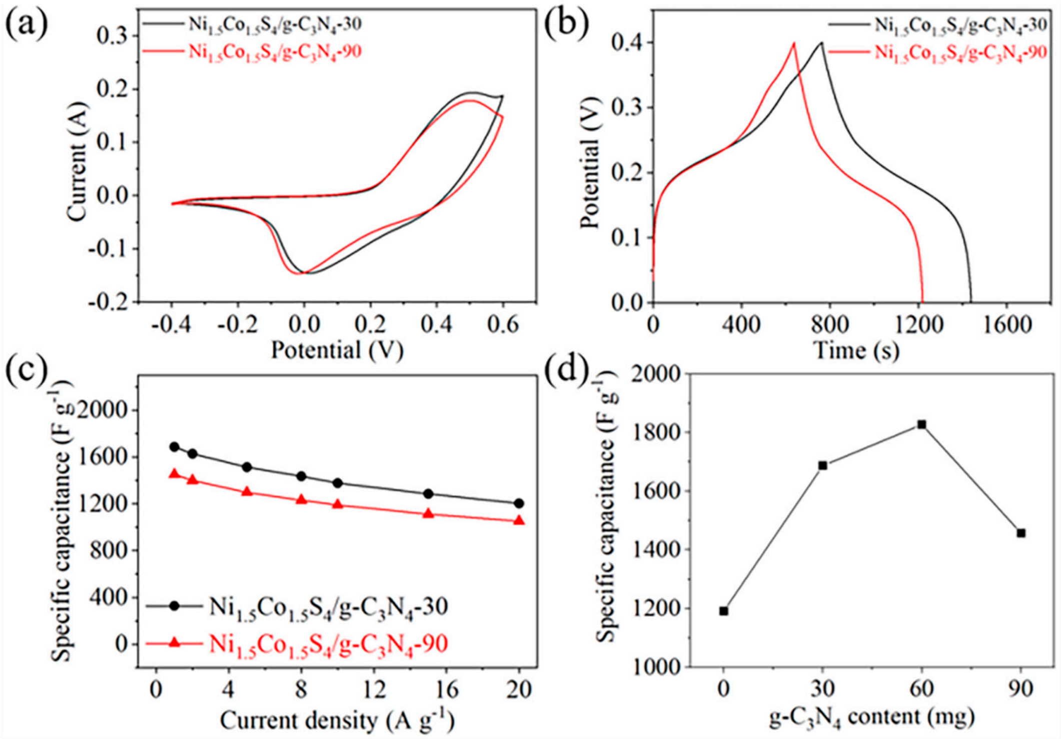

Figure S4 Electrochemical properties of the $Ni_{1.5}Co_{1.5}S_4$/g-C_3N_4 with different content of g-C_3N_4: (a) CV curves, (b) GCD curves, (c) the specific capacitance at different current densities, and (d) the dependence of specific capacitance on g-C_3N_4 content.

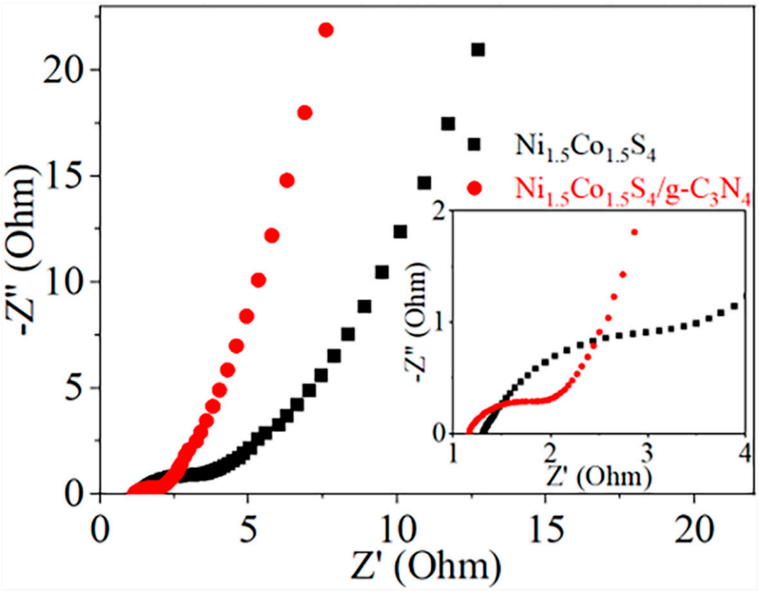

Figure S5 Nyquist plot of pure $Ni_{1.5}Co_{1.5}S_4$ and $Ni_{1.5}Co_{1.5}S_4$/g-C_3N_4

Table S1. The cumulative pore volume of $Ni_{1.5}Co_{1.5}S_4$ and $Ni_{1.5}Co_{1.5}S_4$/g-C_3N_4

Sample	Mesopore pore volume (cm^3/g)	Macropore Pore Volume (cm^3/g)
$Ni_{1.5}Co_{1.5}S_4$	0.04	0.06
$Ni_{1.5}Co_{1.5}S_4$/g-C_3N_4	0.07	0.05
